# Feasibility and diagnostic power of transthoracic coronary Doppler for coronary flow velocity reserve in patients referred for myocardial perfusion imaging

**DOI:** 10.1186/1476-7120-6-12

**Published:** 2008-03-29

**Authors:** Eva Maret, Jan Engvall, Eva Nylander, Jan Ohlsson

**Affiliations:** 1 Department of Clinical Physiology, Ryhov County Hospital, SE-551 85 Jonkoping, Sweden; 2 Department of Clinical Physiology, Linkoping University Hospital, SE-581 85 Linkoping, Sweden

## Abstract

**Background:**

Myocardial perfusion imaging (MPI), using single photon emission computed tomography (SPECT) is a validated method for detecting coronary artery disease. Transthoracic Doppler echocardiography (TTDE) of flow at rest and during adenosine provocation has previously been evaluated in selected patient groups. We therefore wanted to compare the diagnostic ability of TTDE in the left anterior descending coronary artery (LAD) to that of MPI in an unselected population of patients with chest pain referred for MPI. Our hypothesis was that TTDE with high accuracy would identify healthy individuals and exclude them from the need for further studies, enabling invasive investigations to be reserved for patients with a high probability of disease.

**Methods:**

Sixty-nine patients, 44 men and 25 women, age 61 ± 10 years (range 35–82), with a clinical suspicion of stress induced myocardial ischemia, were investigated. TTDE was performed at rest and during adenosine stress for myocardial scintigraphy.

**Results:**

We found that coronary flow velocity reserve (CFVR) determined from diastolic measurements separated normal from abnormal MPI findings with statistical significance. TTDE identified coronary artery disease, defined from MPI, as reversible ischemia and/or permanent defect, with a sensitivity of 60% and a specificity of 79%. The positive predictive value was 43% and the negative predictive value was 88%. There was an overlap between groups which could be due to abnormal endothelial function in patients with normal myocardial perfusion having either hypertension or diabetes.

**Conclusion:**

TTDE is an attractive non-invasive method to evaluate chest pain without the use of isotopes, but the diagnostic power is strongly dependent on the population investigated. Even in our heterogeneous clinical cardiac population, we found that CFVR>2 in the LAD excluded significant coronary artery disease detected by MPI.

## Background

Coronary artery disease (CAD) is the most common cause of death in the western world, despite advances in medical, interventional and surgical treatments. The diagnosis of coronary disease is often based on patient symptoms and an exercise test. However, the sensitivity for the detection of ischemia using exercise in combination with electrocardiography is low, in the range of 60% [1]. Myocardial perfusion imaging (MPI) in combination with stress increases sensitivity to about 85% without compromising specificity [2]. If verification bias (where the selection process results in a higher prevalence of disease than in a clinical population) is taken into account, exercise stress testing as well as other non-invasive testing could have a sensitivity as low as 40% and specificity of 80% [3,4]. The drawback of scintigraphy is exposure to radiation. Echocardiography is inexpensive, widely available, non-invasive and, in combination with Doppler, flow velocity in the epicardial coronary vessels can be interrogated [5]. The method is demanding for the operator but could become a widely available and inexpensive diagnostic modality, especially if combined with pharmacological stress [6-8].

Gould et al have shown that resting coronary blood flow does not decrease until coronary artery diameter is reduced by 85%. The coronary flow reserve, however, begins to decrease already with a 30–45% reduction of the arterial diameter [9,10]. In healthy coronary arteries, adenosine provokes an increase in coronary flow 3–6 times the resting value [11]. Coronary flow velocity reserve (CFVR) is defined as the ratio between coronary flow velocities during maximal hyperaemia and at rest. CFVR has been used as an index of normality in studies of pathological processes such as coronary obstructive disease and endothelial dysfunction [12,13]. Previous studies [14-18] have produced encouraging results for the diagnosis of significant coronary stenoses. Since the method so far has been applied mainly in selected patient populations, we wanted to study if it could be applied in a routine clinical setting, where patients with coronary artery disease as well as with various cardiovascular conditions that are associated with endothelial dysfunction present with chest pain. Thus, in contrast to most previous studies [19-21], patients with previous revascularization, surgical valve procedures, non-sinus rhythm, artificial pacemaker, bundle branch block, hypertension, cardiomyopathy and diabetes mellitus were also included. In this clinical population we set forth to assess transthoracic coronary Doppler echocardiography (TTDE) compared to our standard MPI method.

## Methods

### Study population

Sixty-nine patients (44 men, 25 women; mean age 61 ± 10 years) were enrolled between November 2004 and October 2005. They were all referred for myocardial perfusion imaging because of suspected or known CAD. Eighteen had a history of a previous myocardial infarction and 14 were revascularised (9 with PCI and 5 with CABG). One patient had an aortic valve prosthesis. Sixty-six patients were in sinus rhythm, 2 in atrial fibrillation and one had a pacemaker. Twenty-three patients had a normal electrocardiogram (ECG). Twenty-two patients had anti-hypertensive treatment and 11 were diabetics. Forty-six patients were on betablockers, 10 on calcium inhibitors and 16 on ACE inhibitors. No changes in medication were made prior to the study. Exclusion criteria were acute myocardial infarction, unstable angina, 2^nd ^degree AV-block or higher, obstructive pulmonary disease or treatment with dipyridamole as well as theophylline preparations. The subjects were instructed to abstain from xanthine-containing food and drinks (chocolate, cola, coffee and tea) for at least 24 hours before the study.

The study was approved by the Regional Ethics Committe at Linköping University. All subjects gave informed consent.

### Adenosine infusion protocol

Adenosine is a potent vasodilatator producing maximal coronary vasodilatation within 40–50 seconds. The plasma half-life is less than 10 seconds and side-effects (mainly hyperpnea and flush) and hyperaemia thus subside quickly after termination of infusion. Adenosine was administered intravenously (0.140 mg/kg/min) for 5 minutes. A 12-lead ECG was continuously recorded before, during and up to 5 minutes after the adenosine infusion. Blood pressure was determined at rest and at 1–2 minutes intervals during the infusion. Preset criteria for reducing or stopping the infusion were acute bronchospasm, advanced AV block, decrease in systolic blood pressure > 20 mmHg or patient refusal.

### Transthoracic Doppler echocardiography

**Figure 1:**
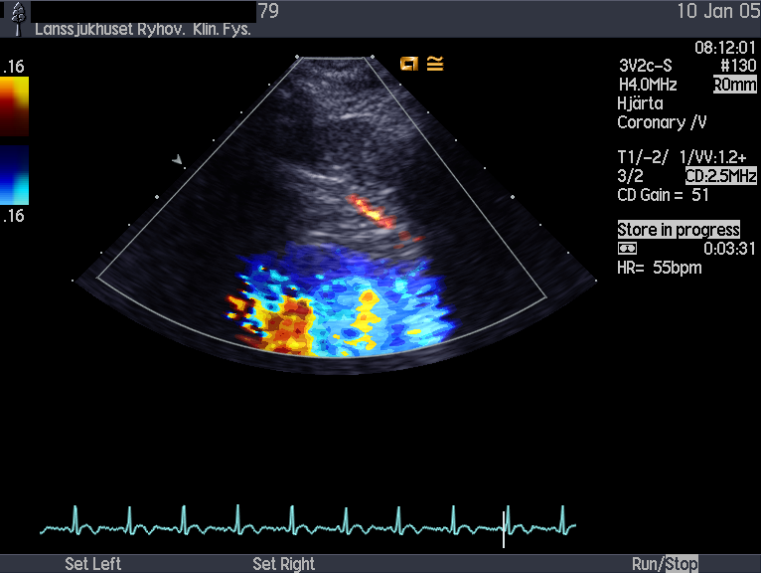
Colour Doppler recording of LAD flow at rest.

**Figure 2:**
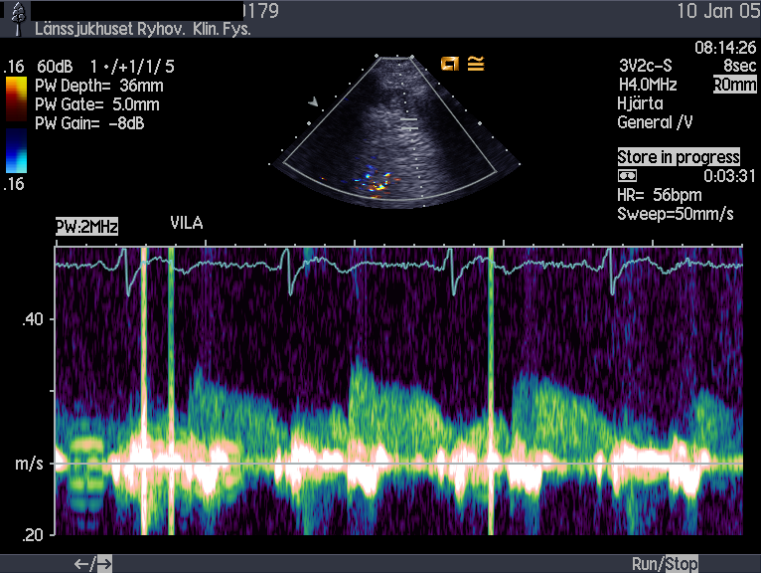
Spectral Doppler recording of LAD flow at rest.

**Figure 3:**
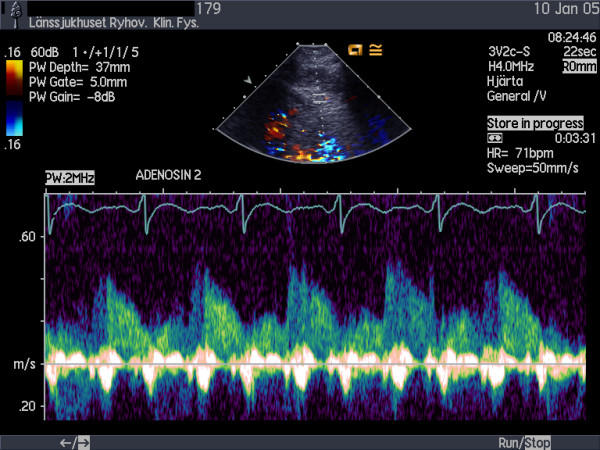
Spectral Doppler recording of LAD flow during stress.

Echocardiographic examinations were performed with a Sequoia C256 or 512 (Siemens Acuson, Mountain View, California) using a broadband transducer (3V2c and 4V1c respectively). For colour Doppler (CD) flow mapping, the velocity range was set at 12 to 20 cm/s. The colour gain was adjusted to provide optimal images with minimal "bleeding" of colour onto tissue, fig 1. Coronary flow velocity was measured with pulsed wave Doppler at 3.5 MHz using CD as a guide. Gate size was set at 4–5 mm. Angle correction was performed if the angle between the CD flow and the Doppler beam exceeded 20 degrees and was maintained during rest and stress studies. The spectral trace of the coronary flow was characteristically biphasic with a dominating diastolic component. Stop frames and clips were digitally recorded for off-line analysis, figs 2 and 3.

The patients were examined in the left lateral position. The aim was to record flow in the most distal part of the left anterior descending artery (LAD). To image the distal LAD, the transducer was positioned either at the cardiac apex or one intercostal space above and focused on the proximal field. The transducer was rotated and tilted until the distal coronary segment could be visualized by CD at the epicardial part of the anterior wall. Alternatively, a short axis view of the left ventricular apex and anterior groove was interrogated with CD. When diastolic CD blood flow was detected, the transducer was slowly rotated clockwise to obtain the best long axis view of the LAD.

If no CD blood-flow from the LAD was visualized in the baseline condition before the infusion, a new resting recording was made at least 5 minutes after the infusion was stopped. No contrast agent was used.

Stop frames of the spectral Doppler signal were repeatedly stored during the investigation. Each study was analysed off-line. Measurements were performed by tracing the contour of the Doppler signal on the ultrasound monitor. Signals with a well defined outline were analysed and diastolic peak velocities, mean diastolic velocities and diastolic velocity time integrals were measured at baseline and peak hyperaemic conditions. Due to systolic movements of the heart, the systo-diastolic averaged peak velocity and velocity time integral were not always determined. The measurements were, if possible averaged over three consecutive heart beats. Coronary flow reserve was calculated as the ratio of peak hyperaemic and basal mean diastolic velocities without knowledge of the results of the SPECT myocardial perfusion imaging.

### Myocardial perfusion imaging

Gated myocardial SPECT was performed using a two-day stress/rest protocol. Adenosine was infused continuously for 5 minutes. At 3 minutes into the infusion, Tc-99m tetrofosmin (5.7 MBq/kg bodyweight) was injected. Supine gated SPECT images were acquired 45–60 minutes later. Rest images were obtained 3–5 days later using 8.6 MBq 99mTc- tetrofosmin/kg bodyweight with acquisition starting 45–60 minutes after the injection. The patients were offered cold water to drink before acquisition in order to diminish bowel artifacts.

The acquisitions were made on a dual-detector gamma camera (ECAM Siemens Medical Systems Inc) with a low energy high resolution collimator using 64 projections over 180° (right anterior oblique 45° to left posterior oblique 45°), 30 s per projection. A 19% window was asymmetrically placed (129–155 keV) on the 140 keV peak, asymmetry 2%. The gated and un-gated data were separately reconstructed on a Hermes Medical Solutions (Stockholm, Sweden) workstation. Prefiltering with a Butterworth filter (cut off 0.8/cm, order 10) was applied followed by filtered back projection. No scatter or attenuation correction was applied. The reconstructed transaxial data were realigned along the heart axis. The short axis slices were then processed with the automatic software packages QPS and QGS (Cedars-Sinai Medical Center, Los Angeles, CA, USA).

The images were interpreted separately by 2 experienced physicians without knowledge of the outcome of the TTDE. Each observer classified the studies as normal, probably normal, equivocal, probably abnormal and abnormal. In case of an equivocal result, the gated study was evaluated in cine mode, making it possible to reclassify the study as either probably normal or probably abnormal. Finally, the normal and probably normal studies were grouped together as were the probably abnormal and the abnormal studies. The perfusion defects were also assigned to LAD, LCX and/or RCA territories. In three cases there was disagreement between the two observers and consensus was negotiated.

The "Summed Stress Score" as a measure of perfusion abnormalities was also quantitatively analysed with the software package commonly used in our clinic (QPS Cedars Sinai, Los Angeles, CA, USA).

### Statistical analysis

Descriptive statistics were used in combination with Students' t-test, paired or un-paired, when appropriate as well as Pearson Chi-square (SPSS Inc, Chicago, Illinois, USA). A p-value of < 0.05 was considered significant.

## Results

The clinical characteristics of the patient population are summarized in table 1.

**Table 1:** Clinical characteristics. Demographic data and clinical characteristics. When appropriate, mean values ± SD are given.

	Total	Successful CFVR	Unsuccessful CFVR	Sign.
Number of patients	69	48	21	
Age (year)	61 ± 10	61 ± 11	60 ± 10	ns
Gender (Male/Female)	44/25	34/14	10/11	ns
BMI	27 ± 4	27 ± 4	28 ± 4	ns
BPsys (mmHg)	133 ± 19	134 ± 19	133 ± 22	ns
BPdia (mmHg)	76 ± 9	76+13	76 ± 6	ns
Diabetics (number)	11	9	2	ns
CAD (number)	22	16	6	ns
Normal EKG (number)	23	18	5	ns

The outcome in groups where CFVR was successfully calculated is summarized in table 2. TTDE identified coronary artery disease, defined from MPI as reversible ischemia and/or permanent defect, with a sensitivity of 60% and a specificity of 79%. The positive predictive value was calculated to 43% and the negative predictive value to 88%. Due to few patients with abnormal SPECT, the pre-test-probability was only 21%. The mean coefficient of variation for the calculation of CFVR, obtained by one operator (intraobserver variability), was 3.9%.

**Table 2:** Comparison between CFVR and MPI. CFVR and result of MPI in groups where CFVR was successfully calculated (p = 0.043, Chi-square)

CFVR/Scint	Normal MPI	Abnormal MPI	Total
>2	30	4	34
≤2	8	6	14
Total	38	10	48

**Figure 4:**
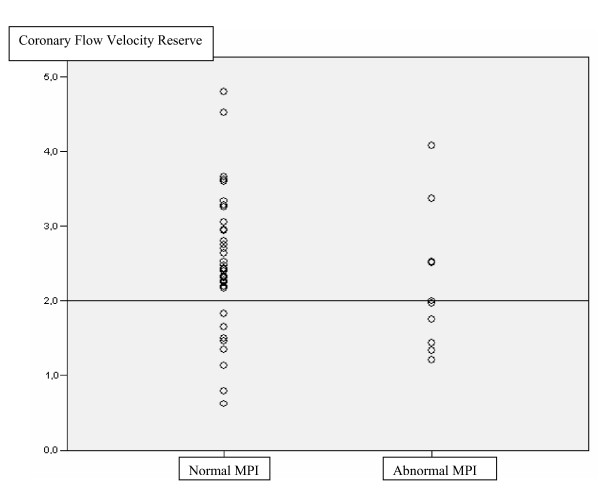
**Individual results of CVFR plotted against result from MPI.** Heavy line indicates the limit for CFVR at 2.

Fig 4 shows the spread in individual CFVR values for patients with normal or abnormal MPI. The mean value for patients with normal MPI was 2.51 and for abnormal MPI 2.22, p = 0.36. The four patients shown in figure 4 having abnormal SPECT but CFVR>2 had spectral Doppler signals of good quality. Two of these patients had perfusion defects in territories of the right coronary artery (RCA) but not the LAD where CFVR was measured. In the third patient, the Doppler signal drifted into a stent in the LAD during stress giving a higher stress velocity and therefore a falsely high CFVR. The fourth patient had a stenosis in the anastomosis between the left internal mammary artery (LIMA) and the LAD causing an overestimation of CFVR.

The eight patients with normal SPECT but reduced CFVR all had risk factors for endothelial dysfunction.

No serious adverse reactions occurred during adenosine infusion. In 5 patients, the hyperpnea and discomfort caused patient movements precluding the Doppler recording but the adenosine-infusion was not disrupted. TTDE without contrast visualized a distal segment of the LAD in 52 of the 69 patients but CFVR could be calculated only in 48 patients (success rate 70%).

No significant differences were found regarding age, gender, BMI, systolic blood pressure, known CAD (previous AMI, PCI and/or CABG), normal/abnormal myocardial SPECT between the group where CFVR was successfully calculated and the group where CFVR was not obtained. Also, the groups did not differ with reference to ischemic burden according to the "Summed Stress Score" calculated with QPS, (4.8 ± 5.4 and 6.7 ± 7.0 respectively, p = 0.27).

## Discussion

A CFVR >2 identified patients without SPECT signs of fixed or reversible coronary artery disease with a moderately high specificity (79%).

Our study population contained patients with previous revascularization, hypertension, cardiomyopathy and diabetes mellitus. These conditions are associated with reduced endothelial function that might obscure the detection of coronary artery disease [22], thus explaining the low sensitivity (60%). In addition, patients with LBBB/pacemaker/non-sinus rhythm and previous valve procedures were also included. Most previous validation studies of TTDE have not had this approach to patient selection [19-21]. The low pre-test-probability in our study (21% according to MPI) also influenced the sensitivity of transthoracic coronary Doppler.

Since MPI is the most frequently used non-invasive method for ruling out significant coronary artery disease and its high diagnostic accuracy is well documented [23,24], we chose to use it as our reference method, despite its rather low specificity [25]. A drawback with scintigraphy is the radiation exposure, about 10 mSv in our clinic. Invasive coronary angiography was not clinically indicated in our patient population and could therefore not be used as reference.

Which conditions may complicate the use of TTDE? Adenosine, a strong dilator of the coronary microcirculation, is considered to have little effect on the epicardial arteries [26]. If the epicardial vessel diameter is unchanged during the infusion of adenosine, velocity can be used as a surrogate for flow, mainly reflecting the condition of the microvascular circulation. However, Kiviniemi et al have shown that the epicardial coronary arteries increase in diameter up to 31% during adenosine infusion [27]. The measurement of flow velocity increase can therefore underestimate the increase in volume flow and thereby the increase in coronary flow reserve.

Withdrawal of vasoactive medication was not required in our clinical routine. However, betablockers have been shown to increase CFVR while vasodilators have caused an underestimation of CFVR by reducing the maximal vasodilator response to adenosine[28]. It has been suggested that anti-anginal medication should be withheld 48 hrs prior to vasodilator stress [29].

In previous studies, adenosine or dipyridamole were used as vasodilators [30]. We chose adenosine because of our familiarity with its properties. The short half life enabled the recording of resting values after the termination of adenosine infusion, when necessary. However, the hyperpnoea associated with the vasodilating effect of adenosine prevented us from performing wall motion analysis and it caused such discomfort that calculation of CFVR was impossible in some patients.

We have refrained from using external ultrasound contrast due to signal scattering in colour as well as in spectral Doppler recordings. Recent publications have shown that the use of ultrasound contrast markedly improves the measurement of coronary flow velocity [31-33]. However, in view of recent warnings issued by FDA, the use of echo contrast should be considered with caution [34]. Better ultrasound hardware, e.g. more channels, increased colour Doppler sensitivity and better focused ultrasound probes could further improve future results.

This study was performed by one experienced ultrasound operator, who in preparation for this study, devoted considerable time to master the skills of coronary scanning. Still, the success rate was low, 70%, partly due to difficult scanning conditions in overweight patients and partly due to the discomfort induced by adenosine itself. Thus, the selection of patients seems as important as training in improving the success rate. In this study, we limited TTDE scanning to the LAD and the result was compared with MPI in the LAD territory. However, other authors have shown the applicability of scanning all three coronary vessels [35].

## Conclusion

In conclusion, based on our results, we suggest that CFVR>2 in the LAD will exclude significant coronary artery disease in a clinical setting. A low CFVR is more difficult to interpret since multiple cardiovascular and metabolic risk factors as well as epicardial vessel disease or microvascular dysfunction might be responsible. This complicates the use of TTDE as a method for detecting coronary disease in individual patients with a heterogeneous clinical background. However, because of its non-invasive nature and absence of radiation, the method seems to be well suited for repeated investigations of CFVR in a variety of pathophysiological conditions, such as detecting changes in endothelial function after intensified treatment in patients with cardiovascular risk factors. These results may stimulate future clinical studies on the use of TTDE in clinical practice.

## Competing interests

The author(s) declare that they have no competing interests.

## Authors' contributions

EM planned the study, investigated all patients, performed all measurements and the main part of writing the manuscript. JE participated in the planning of the study, statistical analysis and in the writing process. JO participated in the planning of the study, was responsible for the evaluation of the scintigraphic part and took part in statistical analysis and writing the manuscript. EN was involved mainly in evaluation of the results and in the writing and editing process. All authors have read and approved the final manuscript.
